# The association between hope, marital status, depression and persistent pain in men and women following cardiac surgery

**DOI:** 10.1186/s12905-017-0501-0

**Published:** 2018-01-02

**Authors:** Ann Kristin Bjørnnes, Monica Parry, Irene Lie, Ragnhild Falk, Marit Leegaard, Tone Rustøen

**Affiliations:** 1 Department of Research and Development, Division of Emergencies and Critical Care, Oslo University Hospital, Ullevål, P.O Box 4956, Nydalen, 0424 Oslo, Norway; 2 Lawrence S. Bloomberg Faculty of Nursing, University of Toronto, 155 College Street, Suite 130, Toronto, ON M5T 1P8 Canada; 3 Center for patient centered heart- and lung research, Department of Cardiothoracic Surgery, Division of Cardiovascular and Pulmonary Diseases, Oslo University Hospital, Ullevål, P.O Box 4956, Nydalen, 0424 Oslo, Norway; 4 Oslo Centre for Biostatistics and Epidemiology, Research Support Services, Oslo University Hospital, Ullevål, P.O Box 4956, Nydalen, 0424 Oslo, Norway; 5 Faculty of Health Sciences, Institute of Nursing, Oslo and Akershus University College of Applied Sciences, P.O Box 4, St. Olavs Plass, N-0130 Oslo, Norway; 6 Institute of Health and Society, Oslo University Hospital, Ullevål, P.O Box 4956, Nydalen, 0424 Oslo, Norway

**Keywords:** Cardiac surgery, Hope, Marital status, Persistent pain, Depression

## Abstract

**Background:**

Cardiac surgery is a major life event, and outcomes after surgery are associated with men’s and women’s ability to self-manage and cope with their cardiac condition in everyday life. Hope is suggested to impact cardiac health by having a positive effect on how adults cope with and adapt to illness and recommended lifestyle changes.

**Methods:**

We did a secondary analysis of 416 individuals (23% women) undergoing elective coronary artery bypass graft and/or valve surgery between March 2012 and September 2013 enrolled in randomized controlled trial. Hope was assessed using The Herth Hope Index (HHI) at three, six and 12 months following cardiac surgery. Linear mixed model analyses were performed to explore associations after cardiac surgery between hope, marital status, depression, persistent pain, and surgical procedure.

**Results:**

For the total sample, no statistically significant difference between global hope scores from 3 to 12 months was observed (ranging from 38.3 ± 5.1 at 3 months to 38.7 ± 5.1 at 12 months), and no differences between men and women were observed at any time points. However, 3 out of 12 individual items on the HHI were associated with significantly lower scores in women: #1) *I have a positive outlook toward life*, #3) *I feel all alone*, and #6) *I feel scared about my future*. Over the study period, diminished hope was associated with older age, lower education, depression prior to surgery, and persistent pain at all measurement points. Isolated valve surgery was positively associated with hope. While neither sex nor marital status, as main effects, demonstrated significant associations with hope, women who were divorced/widowed/single were significantly more likely to have lower hope scores over the study period.

**Conclusion:**

Addressing pain and depression, and promoting hope, particularly for women living alone may be important targets for interventions to improve outcomes following cardiac surgery.

**Trial registration:**

Clinical Trials gov Identifier: NCT01976403. Date of registration: November 28, 2011.

## Background

Approximately 8% of men and 5% of women in the United States (US) have coronary heart disease (CHD), and 2.5% of the US population have heart valve disease, with similar prevalence in both men and women [1]. Complex surgical procedures (e.g., coronary artery bypass graft [CABG] surgery and heart valve surgery) are commonly performed to reduce cardiac mortality and to relieve associated symptoms such as cardiac pain [2]. Cardiac surgery is a major life event [3], and outcomes after surgery are associated with the individual’s ability to self-manage and cope with their cardiac condition in everyday life. Following surgery, individuals are encouraged to set and achieve goals related to modification and control of physiological risk factors (e.g., weight, blood pressure, blood lipids) and promotion of healthy lifestyle routines related to nutrition, exercise and sleep [4]. Hope refers to a goal-directed cognitive process, including pathway thinking (i.e., the capacity to establish ways for reaching goals) and agency thinking (i.e., ability to sustain the motivation to purse goals) [5]. In that way hope might be an important factor after cardiac surgery [6].

Hope is also a positive phenomenon, and is linked to optimism [7], psychological well-being, improved physical health and positive CHD outcomes [6, 8, 9]. Dufault and Martocchio (1985) propose an often cited definition [10] that suggest hope is “a multidimensional dynamic life force characterized by a confident yet uncertain expectation of achieving a future good which, to the hoping person, is realistically possible and personally significant”. Hope is future-oriented, and has a positive association with psychosocial relationships [5, 11, 12], stimulates coping, and adaptation to life changes [5, 13] across affective, cognitive, and behavioural dimensions [11, 14]. Hope is described to be negatively correlated with low socioeconomic status, low education, living alone, and other chronic conditions [15, 16]. Low levels of hope are also reported to be related to persistent pain, fatigue, lack of control, impaired cognition, unresolved grief, undesirable living arrangements and poor health-related quality of life (HRQL) across a wide range of conditions [16–18]. DuBois et al. [19] suggest that positive psychological constructs, such as hope, impact cardiac health by having a positive effect on how adults cope with and adapt to illness and recommended lifestyle changes. In a prospective cohort study by Feldman et al. [6] (*N* = 391), hope predicted health-promoting behaviours at one-month follow-up in immigrants undergoing cardiovascular risk screening and assessment. Similarly, Dunn et al. [12] (*N* = 324) found that individuals with CHD who experienced diminished hope were less likely to exercise after coronary events.

In contrast to hope, depression is associated with poorer recovery and outcomes after cardiac surgery [3, 20]. Approximately 4% of the general US adult population suffers from major depression [20]. The prevalence is about twice as high in women compared to men, and depression is associated with a 50% increased risk of cardiac events in women [21]. Preoperative depression is predictive of post-surgery depression and is associated with decreased cardiac symptom relief, quicker return of ischemic symptoms, more frequent rehospitalisation, poorer HRQL and increased mortality in the immediate postoperative period [20, 22]. Approximately 20% of patients who had CABG surgery remained depressed beyond the immediate postoperative recovery period [23]. When merging results from 26 studies including 4023 participants, Alarcon et al. [24], found that depression had a significant negative correlation with hope (mean *p −* 0.52, *p* < 0.05).

Unrelieved acute pain is associated with an increased risk for persistent pain, with approximately 10 to 50% of acute pain transitioning into persistent pain [25, 26]. In a prospective Canadian study [27] (*N* = 1247), 10% of respondents continued to report persistent postoperative pain up to two years following cardiac surgery. Women have a higher prevalence of clinically relevant pain and report more persistent pain of moderate to severe intensity up to two years after cardiac surgery [27–29]. Interestingly, persistent pain disorders have not been associated with lower levels of hope in the general population [30], however; the association between hope and persistent pain after cardiac surgery including differences related to sex has not been investigated. Sex differences are particularly important, since there is a persistent gap in the representation of women in cardiovascular research [31], and sex-stratified data reporting is recommended [21].

The Herth Hope Index (HHI) is a well-established measure of hope [14], designed to measure a global, time-independent sense of hope [32]. The HHI is based on the global and the specific dimensions of hope conceptualized by Dufault and Martocchio [10]. Although the HHI has been used to measure hope in different populations, the majority of studies have been related to cancer, and only one study has assessed hope in the general population [30]. Ten studies have used the HHI to assess hope in the cardiac population [7, 18, 33–40]. Combined, these ten studies included only 734 respondents and due to cross-sectional designs, small sample sizes, and variable follow-up times, the evidence is inconclusive. No study found was investigating the role of hope in individuals after isolated heart valve surgery.

### Aims

The aims of this study were to: 1) describe hope in men and women after cardiac surgery, and 2) to examine the association between hope after cardiac surgery and the following factors: age, marital status, education, surgical procedure, depression, and persistent pain after cardiac surgery.

## Methods

We did a secondary analysis of 416 individuals (23% women) undergoing elective coronary artery bypass graft (CABG) and/or valve surgery between March 2012 and September 2013 enrolled in randomized controlled trial. As no between-group differences were found in the main trial the whole sample was included in the present study. Study procedures have been described in details elsewhere [41]. Briefly, eligible participants were ≥18 years old, able to speak, read and understand Norwegian, scheduled to elective CABG and /or valve surgery, and were able to care for themselves post-discharge. The participants were recruited from two, separate cardiothoracic surgical units at Oslo University Hospital, Norway. Data collection was carried out prior to surgery, and at three, six and 12 months post-surgery. The Regional Committee for Medical Research Ethics in Eastern Norway approved the research and all participants provided written informed consent.

### Measures

Hope was assessed at three, six and 12 months post-surgery, and was measured using the Norwegian version of the *Herth Hope Index* (HHI). The HHI is a shortened version of Herth Hope Scale [32], and contains 12 items measured on a 4-point Likert scale ranging from 1 (strongly disagree) to 4 (strongly agree). The scale gives one global score that ranges from 12 to 48, as well as single-item scores that range from 1 to 4 with scores in the upper range indicating greater levels of hope. The HHI has been validated in a sample of 1893 Norwegian adults form the general population with an internal consistency coefficient of 0.81 (Cronbach’s alpha) [42], and in the current sample, we obtained a Cronbach’s alpha between 0.85 (3 months) and 0.86 (12 months after surgery).

Previous research has shown that marital/cohabitant status is a valid proxy for social support [43, 44], and we obtained information on marital status and living conditions from the demographic form prior to surgery. All respondents were asked to indicate if they were living alone or living with somebody (i.e., living with a spouse/partner, living with other adults, divorced, widowed or single).

Depression as a comorbid condition prior to surgery was assessed using *The Self-Administered Comorbidity Questionnaire* (SCQ-16) [45]. The SCQ-16, which has obtained a pre-test reliability of 0.94 [45], includes 15 common medical conditions, including depression, and one optional item. Respondents were asked to indicated if they had one of the listed medical conditions by answering yes/no or fill out the optional item.

Persistent postoperative pain is defined by the convention of the International Association for the Study of Pain as pain developing after surgery, with other causes of pain excluded and persisting for at least two to three months after surgery [46]. Persistent pain intensity was obtained using an item from the *Brief Pain Inventory Short Form* (BPI-SF) [47] at three, six and 12 months post-surgery. Data was captured as worst pain intensity in the last 24 h using a 0 (no pain) to 10 (pain as bad as you can imagine) numeric rating scale. The BPI-SF is a reliable and valid scale for assessing acute and persistent pain in individuals after cardiac surgery, and obtained a Cronbach’s alpha coefficients between 0.84 and 0.94 in the Norwegian validation study [48].

### Analysis

Data were were analyzed using the Stata statistical software [49]. All tests were two-tailed and associations were considered statistically significant if *p* ≤ 0.05. The distribution of data was assessed graphically (i.e., histograms), depending of normality, continuous variables were compared using a Student’s *t*-test or Man-Whitney *U*–test. Differences between categorical variables were assessed using cross tabulation with chi-square analyses or the Fisher’s exact test.

Missing values in the HHI were replaced with the item’s mean value if 20% or less of the items were missing from an individual’s response. If more values were missing, the respondents were not included in the longitudinal analyses. The two negative items (i.e., 3 and 6), were reverse scored so that a higher score would indicate higher levels of hope [32].

Associations between hope (i.e., HHI) and the factors of interest were assessed by estimating a linear mixed model with a correlation structure based on the AIC (Aikaike Information Criterion) [50]. Selected covariates included time after surgery, sex, age, marital status (i.e., married/cohabitant/partner or living alone), education (i.e., primary school, and greater than primary school), surgical procedure (i.e., isolated CABG surgery, isolated valve surgery or both CABG/valve surgery), depression, and persistent pain (worst pain intensity). Marital status and depression have shown significant associations with hope in previous studies among individuals with CHD, whereas age and sex have had a significant impact on hope levels in the general population. Since no previous study has investigated hope after isolated valve surgery, surgical procedure was included as a covariate. Persistent pain intensity was considered as a time-varying factor. The final model assessed the HHI total score as a dependent factor, with time after surgery, age, sex, marital status, surgical procedure, depression and persistent pain (worst pain intensity) as independent factors. Interaction between sex and marital status was included as well. A significant interaction term would imply that the relationship between hope and marital status differed between sexes. The covariates were included as fixed factors in the model, and participants and time after surgery were considered as random factors with an unstructured covariance matrix.

We compared our results with previous studies using means, standard deviations, and sample sizes to calculate effect sizes with 95% confidence intervals (CIs) which were represented by Cohen’s *d*. Effect sizes indicate the magnitude of differences between the mean HHI global score at 12 months obtained in our study and the mean HHI global scores obtained from previous studies using the HHI in the cardiac population. Positive effect sizes would suggest that individuals in our study reported higher levels of hope than previous studies, whereas negative effect sizes would suggest lower levels of hope compared to previous studies. Effect sizes <0.2 represent a small effect, 0.5 to 0.8 suggest a medium effect, and >0.8 indicate a large effect size [51].

## Results

### Demographic and clinical characteristics.

**Fig. 1:**
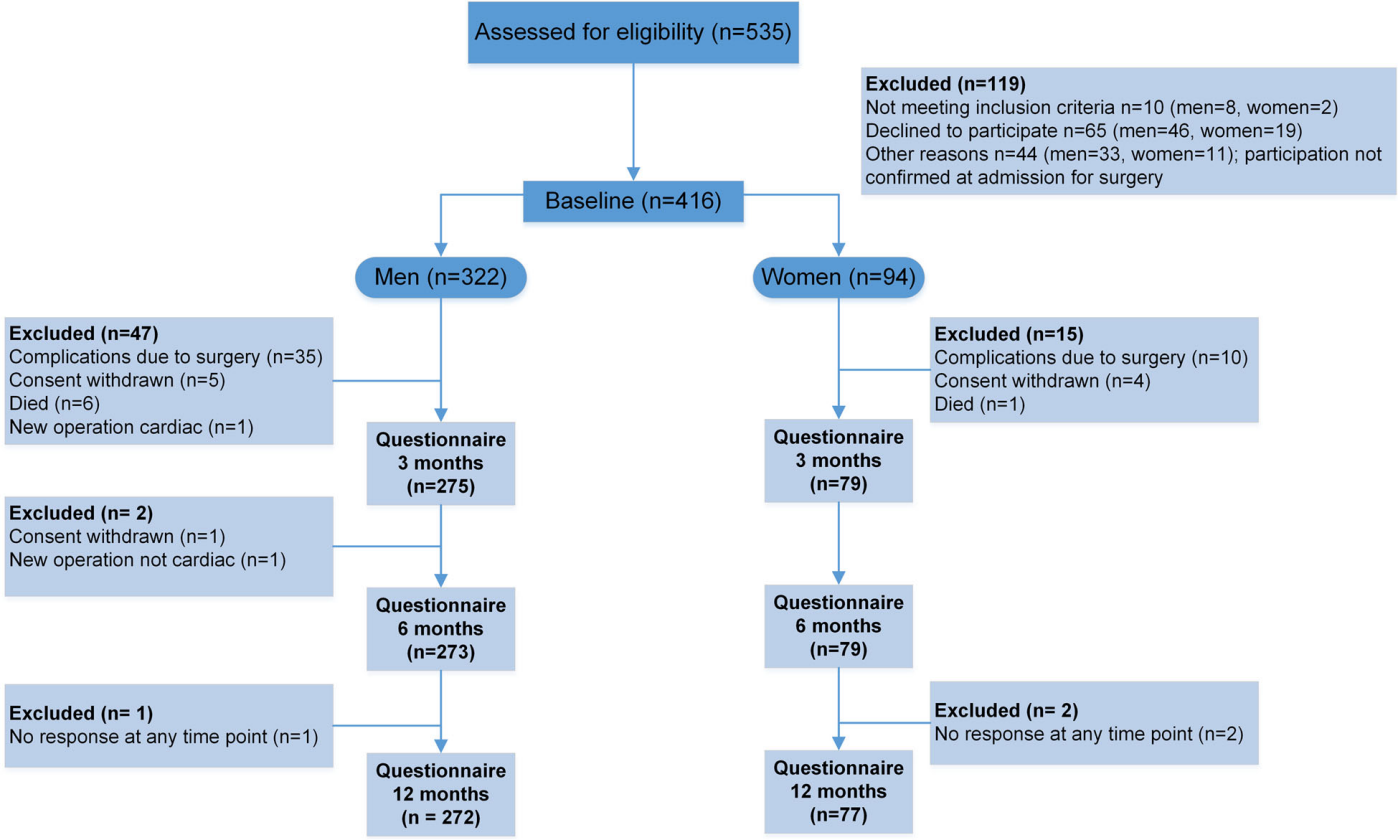
Participant flow through the study

Of the 416 participants at baseline, 349 (84%) completed the study (Fig. 1). The mean age of the participants was 66 ± 10 years, and the majority was men (77%) (Table 1). Women were in average 5 years older than men, and 27% (*n* = 27) of women compared with 16% (*n* = 46) of men had not completed secondary education (*p* = 0.015). Most men (80%, *n* = 241) were married/living with a partner, however, less than half (46%, *n* = 41) of the women reported to live with somebody. For women, the most common surgical procedure was isolated valve surgery (61%, *n* = 57), and isolated CABG surgery, involving internal mammary artery grafts with additional saphenous grafts, was the most frequent procedure among men (51%, *n* = 165). The median number of additional medical conditions for women was 2 (range 0–6), while the median number of comorbid ailment was 1 (range 0–6) among men. The comorbid conditions with a prevalence of 10% or more in one of the sexes are reported in Table 1. Women had a higher prevalence of depression (13%, *n* = 12 vs. 3% *n* = 9, *p* < 0.001) compared to men. No sex differences were observed for preoperative worst pain intensity; however, women reported significantly more intense worst pain at 3, 6 and 12 months compared to men.

**Table 1:** Demographic and clinical characteristics by sex (*N* = 416)

Characteristics		Men (*n* = 322)	Women (*n* = 94)	*p* value^a^
Age	in years, mean ± SD	65 ± 10	70 ± 12	<0.001
Education^b^	Less than high school/secondary, n (%)	46 (16)	24 (27)	0.015
	High school/secondary or higher, n (%)	245 (84)	64 (73)	
Marital status^c^	Married/Cohabitant/Partner, n (%)	241 (80)	41 (46)	<0.001
	Divorced/Widowed/Single, n (%)	59 (20)	49 (54)	
Type of Surgery	Isolated CABG^d^, n (%)	165 (51)	26 (28)	<0.001
	Isolated Heart Valve, n (%)	116 (36)	57 (61)	
	CABG and Valve, n (%)	41 (13)	11 (11)	
Worst pain intenstity	Pre-surgery, mean ± SD	3.0 ± 2.3	3.3 ± 2.4	0.322
	3 months, mean ± SD	1.4 ± 1.9	1.8 ± 2.7	0.013
	6 months, mean ± SD	1.2 ± 1.7	2.0 ± 2.4	0.031
	12 months, mean ± SD	1.0 ± 1.9	1.7 ± 2.5	0.034
Comorbidities^e,c^	Hypertension, n (%)	144 (48)	45 (50)	0.745
	Back/Neck problems, n (%)	70 (23)	27 (30)	0.201
	Diabetes mellitus, n (%)	49 (16)	14 (16)	0.885
	Osteoarthritis, n (%)	34 (11)	29 (32)	<0.001
	Headache, n (%)	16 (6)	11 (12)	0.025
	Rheumatoid arthritis, n (%)	12 (4)	11 (12)	0.004
	Depression, n (%)	9 (3)	12 (13)	<0.001
	Other medical problems, n (%)	69 (23)	22 (24)	0.675
Number of comorbidities^e^ median (range)		1 (0–6)	2 (0–6)	0.010
Body mass index	Kg/m^2^, mean ± SD	27.4 ± 3.8	26.4 ± 4.8	0.046

^a^*p* values obtained from students *t-*tests or Mann–Whitney *U*-test (continuous variables) and Pearson Chi-squared tests or Fischer’s exact (dichotomous variables), ^b^Missing = 37, ^c^Missing = 22 for men and 4 for women, ^d^CABG = Coronary artery bypass graft, ^e^SCQ-16 = The self administered comorbidity questionnaire, SD = Standard dividation

### Hope

The mean global HHI score ranged from 38.3 ± 5.1 at 3 months to 38.7 ± 5.1 at 12 months. The differences in global hope score and mean scores on individual items from 3 to 12 months were small and not statistically significant, and only results from 12 months are shown (Table 2). At 12 months women had significantly lower scores on the items 1, 3 and 6 compared to men. According to the mixed model analysis (Table 3), age, lower education, depression, and persistent pain were inversely associated with hope for both men and women. Isolated valve surgery was positively associated with hope. For women only, marital status (i.e., divorced/widowed/single) was associated with lower hope scores over the study period.

**Table 2:** Level of hope at 12 months in individual items in the Herth Hope Index by sex (*N* = 320)

Individual Items	Men	Women	*p* value
	Mean ± SD	Mean ± SD	
1. I have a positive oulook torward life	3.5 ± 0.6	3.2 ± 0.6	0.003
2. I have short and/or long range goals	3.2 ± 0.6	3.1 ± 0.8	0.465
3. I feel all alone^a^	3.6 ± 0.9	3.2 ± 0.8	<0.001
4. I can see possibilities in the midst of difficulties	3.1 ± 0.8	3.2 ± 0.7	0.819
5. I have a faith that gives me comfort	2.4 ± 1.0	2.6 ± 1.0	0.051
6. I feel scared about my future^a^	3.2 ± 0.8	2.8 ± 0.9	0.004
7. I can recall happy/joyful times	3.5 ± 0.5	3.5 ± 0.6	0.802
8. I have a deep inner strength	3.2 ± 0.6	3.2 ± 0.6	0.926
9. I am able to give and receive caring/love	3.4 ± 0.5	3.4 ± 0.6	0.710
10. I have a sense of direction	3.2 ± 0.6	3.1 ± 0.6	0.502
11. I belive that each day has a potential	3.3 ± 0.6	3.2 ± 0.6	0.285
12. I fell my life has value and worth	3.4 ± 0.6	3.3 ± 0.6	0.243
Herth Hope Index total score	38.9 ± 4.9	38.1 ± 5.9	0.455

Scores range from 1 to 4 with higher scores indicating higher levels of hope

^a^Item is scored reversed

**Table 3:** Linear mixed model; variables associated with the Herth Hope Index total score^a^ across time

Variables	Coef.	95% C.I	*p* value
Time^b^			
6 months	- 0.11	[− 0.58, 0.37]	0.646
12 months	0.27	[− 0.21, 0.75]	0.267
Sex^c^	0.94	[− 0.53, 2.42]	0.211
Age > 65^d^	−0.96	[− 1.86, −0.05]	0.039
Surgery^e^			
Isolated Valve	1.16	[0.23, 2.11]	0.014
Valve and CABG	0.29	[− 1.22, 1.81]	0.711
Marital status^f^	−0.79	[− 2.01, 0.43]	0.208
Educational status^g^	1.21	[0.09, 2.31]	0.033
Depression	−4.78	[− 6.71, − 2.83]	<0.001
Worst pain intensity	−0.39	[− 0.54, −0.23]	<0.001
Sex^a^Marital status	−2.38	[− 4.61, − 0.12]	0.039

^a^Higher scores indicating higher levels of hope

^b^Time after surgery, 3 months as reference

^c^Men = 0, Women = 1

^d^Age ≤ 65 = 0, Age > 65 = 1

^e^Isolated CABG surgery as reference

^f^Married/Cohabitant/Partner = 0, Divorced/Widowed/Single = 1

^g^Less than high school/secondary = 0, High school/secondary or higher =1

The mean global HHI scores reported in different studies in the cardiac population and the effect sizes represented by the Cohen’s *d* are reported in Table 4. The results indicated that the current study obtained higher scores on the HHI compared to Madhavi et al. [34] (i.e., large effect), Evangelista et al. [39] (i.e., small effect), Rustøen et al. [38] (i.e., small effect), and Staples et al. [40] (i.e., small effect). The current study obtained lower scores on the HHI compared to Bay et al. [36] (i.e., small effect), Beckie et al. [7] (i.e., medium effect), Eriksson et al. [35] (i.e., medium effect), McGurk et al. [33] (i.e., small effect), Kelly-Tobin [18] (i.e., small effect), and Van Kuiken et al. [37] (i.e., small effect).

**Table 4:** Mean scores and standard deviation reported in different studies that used the Herth Hope Index and standardized effect sizes (Cohen’s *d)*

Study (year), country	Sample (N)	HHI global, mean ± SD	Cohen’s *d* [95% CI]
Present study, Norway	Cardiac surgery (416)	3 months: 38.3 ± 5.1, 6 months: 38.3 ± 5.4, 12 months: 38.7 ± 5.1	0.46 [0.35, 0.57]^a^
Mahdavi (2016), [34] Iran	Cardiac surgery (80)	Pre-surgery: 20.2 ± 8.1 1 month: 23.4 ± 6.8	2.81 [2.51, 3.11]^b^
Kelly-Tobin (2016), [18] USA	Women after MI (91)	39.06 ± 4.65	- 0.32 [− 0.63, − 0.01]^c^
Eriksson (2013), [35] Sweden	After MI (13)	1 month: 40.6 ± 5.8, 13 months: 41.6 ± 5.4, 24 months: 41.5 ± 6.7	- 0.57 [− 1.12, − 0.01]^b^
McGurk (2010), [33] USA	Heart Failure (65)	39.03 ± 5.67	- 0.06 [− 0.32, 0.20]^b^
Bay (2008), [36] Sweden	CABG surgery (166)	Pre-surgery: 40.5 ± 5.0 1 month: 40.9 ± 5.2	- 0.43 [− 0.67, − 0.19]^d^
Van Kuiken (2008), [37] USA	Heart Failure (67)	38.8 ± 7.03	- 0.02 [− 0.3, 0.24]^b^
Rustøen (2005), [38] Norway	Heart Failure (88)	37.69 ± 5.3	0.2 [− 0.04, 0.43]^b^
Evangelista (2003), [39] USA	Women, Heart Transplant Recipients (50)	35.84 ± 5.08	0.19 [− 0.17, 0.56]^c^
Beckie (2001), [11] USA	Women, after cardiac events (93)	40.93 ± 5.34	- 0.59 [− .91, − 0.28]^c^
Staples (1997), [40] Canada	Cardiac surgery waiting period (21)	38.5 ± 4.1	0.04 [− 0.04, 0.48]^b^

^a^Scores at 12 months compared with general Norwegian population (Rustøen et al. 2003), ^b^Compared with values at 12 months present study, ^c^Compared with values for women at 12 months present study, ^d^Values at 1 month control group compared with 12 months present study, CI = Confidence Interval, MI = Myocardial Infarction, CABG = Coronary Artery Bypass Graft surgery

## Discussion

The purpose of this study was to describe hope characteristics and to identify factors that were associated with hope in men and women following cardiac surgery. For the total sample, no statistically significant difference between global hope scores from 3 to 12 months was observed, and no between sex differences were observed at any time points. However, 3 out of 12 items on the HHI were associated with lower scores in women: #1) *I have a positive outlook toward life*, #3) *I feel all alone*, and #6) *I feel scared about my future*. Diminished hope was associated with older age, lower education, depression prior to surgery, and persistent pain (worst pain intensity) at all measurement points. Isolated valve surgery was positively associated with hope. While neither sex nor marital status, as main effects, demonstrated significant associations with hope, women who were divorced/widowed/single were significantly more likely to have lower hope scores over the study period.

The majority of findings related to the HHI in the cardiac population are based on cross-sectional studies in smaller cohorts of individuals, and longitudinal studies are notably lacking. Our results add to the evidence, and at 12 months our global hope average score was lower than that for a Swedish intervention study one month after cardiac surgery [36], and compared to those reported at 13 months in a small longitudinal study by Erikson et al. [35]. Our HHI scores were substantial higher than those reported by Mahdavi et al. [34] one-month post-cardiac surgery in an Iranian sample, and those reported 5 years after heart transplantation [39]. Our higher scores compared to those after heart transplantation [39] may be explained by the severity of the cardiac condition, although other studies assessing hope in individuals suffering from life-threatening diseases have obtained HHI scores similar to our study [42, 52], suggesting that there is no linear relationship between experiences of hope and disease severity. Socioeconomic circumstances are also reported to influence hope [15, 53], and we observed a negative association between lower education and hope. Similarly, the inferior hope outcomes in the Iranian sample [34] may also be explained by the more challenging socioeconomic context in Iran compared to Norway, including the respondent’s younger age (mean 47.5 ± 11), female sex (70%), and the high rate of unemployment (70%).

Our HHI scores were not significantly different from those obtained from three cross-sectional studies with individuals living with heart failure (HF) [33, 37, 38]. A majority of the individuals in these studies were classified as NYHA class III or IV with ejection fraction less than 0.40, and adjustment for sex was lacking. Since no established cut-off exists for the HHI, direct comparison with these samples of individuals with more severe HF is problematic. However, previous studies have consistently found a significant positive association between hope and HRQL [18, 33, 38, 39, 42], indicating that interventions supporting hope in individuals after cardiac surgery may influence treatment goals.

Interestingly, in the general Norwegian population study women reported significantly higher levels of hope than men [30]. In contrast, women in our study reported significantly lower scores than men (Cohen’s *d* = − 0.33 CI: [−0.59, − 0.07]), and the scores in men at 12 months were significantly higher than those among men in the general Norwegian population (Cohen’s *d* = 0.70, 95% CI: [0.57, 0.84]). Since we did not assess hope prior to surgery, we do not know if this difference appeared before or after surgery. Nevertheless, higher levels of hope might indicate that men were more resilient to negative circumstances during recovery since hope is considered to be a resource that helps individuals to set goals and cope with current life circumstances during an illness [12]. In an early study by Everson et al. [54], the lack of hope in men was found to be associated with accelerated CHD progression. Moreover, emotional well-being is reported to enhance recovery by activating the autonomic nervous system and hypothalamic-pituitary-adrenal axis that buffers the impact of stress on the immune and cardiovascular system [55]. It is also plausible that positive emotions and hope have an indirect effect through contributing to positive health behaviours, and social network engagements [6, 53, 56], both associated with improved cardiac health.

In contrast to men, our results indicate that women experienced a global hope score at 12 months similar to the general Norwegian population [30] (Cohen’s *d* = 0.01, 95% CI:[−0.25, 0.24]). Moreover, the global HHI scores in women were significantly lower compared to Beckie et al. [7] who assessed hope in women hospitalized after a cardiac event (i.e., myocardial infarction [MI], isolated CABG surgery, percutaneous coronary interventions) and those reported by Kelly-Tobin [18] in a cross-sectional study investigating hope in 91 women after MI. Our scores were not significantly different from those reported by women five years after heart transplantation (Cohen’s *d* = 0.19, 95% CI: [− 0.17, 0.56]. The lower scores among women may be related to the higher prevalence of persistent pain reported by women in our study. Women reported significantly higher mean worst pain intensity score at 3, 6 and 12 months compared to men. Moreover, all women consistently reported that they had more pain interference with daily activities than men [29], and persistent pain (worst pain intensity) was negatively associated with hope in the linear mixed model analysis. This evidence suggests hope measured by the HHI is influenced by external factors such as physical function and suffering [35].

The statistically significant lower scores in women related to the three HHI items indicates that women experience worse perceived social support and emotional well-being after surgery compared with men. Women’s older age and widowhood may explain why women were more likely to have lower scores on these items compared to men. According to an exploratory factor analysis [57], item #1 represents *positivity*, and items #3 and #6 represent measures of *support loneliness*. These items are interlinked with both affective and cognitive dimensions of hope [32], and lower scores may indicate less motivation or ability to cope and adapt to the self-management needs and activities that are essential for enhancing recovery after cardiac surgery. Items *I fell all alone* (i.e., item 3*)*, and *I feel scared about my future* (i.e., item 6) indicate an absence of social support, and this is verified with the interaction between sex and marital status from our linear mixed model analysis that indicates women living alone have less hope compared to individuals living with a spouse. A close personal relationship is beneficial for individuals undergoing cardiac surgery [58], and living alone has been significantly associated with death or a new functional disability during the first two years after cardiac surgery [59]. Support has been reported to reduce stress-induced physical (e.g., increased sympathetic activation) and/or psychological (e.g., depression, anxiety) responses [60], associated with lower HRQL after cardiac surgery, particularly for women [60, 61].

The item *I have a positive outlook toward life* (i.e., item 1) is related to positivity [49]. In a prospective qualitative study, Olsman et al. [62] (*N* = 29) reported that transitions between hope, despair, and hopelessness were related to changes in physical condition. Kroemeke et al. [63] (*N* = 173) reported that hope of health improvement was associated with coping and protected the participants from an increase of depressive symptoms six months after MI. Our results complement this evidence, and we found a significant association between preoperative depression and dismissed hope after cardiac surgery. Depression is associated with poorer perceived social support, and men and women with lower support one month after a cardiac event had greater risk of angina, lower HRQL, and persistent depressive symptoms and worse adherence to risk factor and lifestyle modification management adherence (i.e., smoking, taking medication, attending cardiac rehabilitation, and diet) [20, 64] [18, 56]. Conversely, hope helps individuals experiencing health challenges attain goals related to CHD self-management behaviours and form new and positive outlooks towards the future [6, 18].

The positive association between isolated valve surgery and hope is not clear. However, isolated valve surgery is considered to be a more complex and high-risk procedure than isolated CABG surgery [65]. It is associated with higher mortality risk, and it is plausible to think that after surviving the surgery these individuals may feel more optimistic and hopeful about their health outcomes. Moreover, most were isolated valve surgery versus combined valve and CABG surgery. Isolated valve surgery may be considered less chronic than CHD, or isolated CABG and combined CABG and valve surgery. Isolated valve surgery is often indicated for bicuspid aortic valves, the most common adult congenital heart defect [66].

### Limitations

In contrast to previous studies, our study contributes with a prospective, longitudinal design, larger sample size, and the results strengthen the evidence of previous research findings related to hope after cardiac surgery. However, the lack of assessment of hope prior to cardiac surgery is a limitation, although hope has been previously assessed in the general Norwegian population. The associations between hope and outcomes after cardiac surgery may also differ depending on the context of the disease and the individuals [16]. Our results indicate that social support impact hope after cardiac surgery, and diminished hope in women living alone was consistent across all our measure points. However, since we did not include a more comprehensive measurement of social support, we cannot draw a definite conclusion about the association between marital status, social support and hope. Similarly, we obtained information about depression from the SCQ-16 [45], prior to surgery. Inclusion of a separate measurement for depression is preferable, however, self-report is a valid source for information about comorbid ailment, and there is sufficient agreement between self-report data and medical record review [67]. As discussed, hope is associated with goal achievement. To better understand how hope may influence outcomes in men and women following cardiac surgery, future research should attempt to assess hope as a predictor of goal achievement related to self-management behaviours to improve cardiac health. Lastly, given the characteristics of this cardiac surgery sample, it is not known to what degree the results generalise to other ethnic groups, and future research should examine how culture and ethnicity are related to hope and outcomes after cardiac surgery.

## Conclusion

In our sample of cardiac surgery patients, we found that pre-operative depression and persistent postoperative pain were associated with diminished hope. The potential difficult experiences of less hope, specifically for women living alone, may result in a more challenging recovery. Addressing depression and pain, and promoting hope through goal attainment, may be important targets for interventions to improve outcomes for men and women following cardiac surgery.

## Abbreviations

AIC: Aikaike Information Criterion
BPI-SF: The Brief Pain Inventory Short Form
CABG: Coronary Artery Bypass Graft surgery
CHD: Coronary Heart Disease
CI: Confidence Intervals
HF: Heart Failure
HHI: The Herth Hope Index
HRQL: Health-Related Quality of Life
MI: Myocardial Infarction
NRS: Numeric Rating Scale
NYHA: The New York Heart Association (NYHA) Functional Classification1 for Chronic Heart Failure
RCT: Randomized Controlled Trial
SCQ-16: the Self-Administered Comorbidity Questionnaire
